# Impact of Work-Related Chronic Low Back Pain on Functional Performance and Physical Capabilities in Women and Men: A Sex-Wise Comparative Study

**DOI:** 10.1155/2022/6307349

**Published:** 2022-03-03

**Authors:** Hashim Ahmed, Kamal Kishore, Priyadarshani Bhat, Ahmad H. Alghadir, Amir Iqbal

**Affiliations:** ^1^Department of Rehabilitation Sciences, College of Applied Medical Sciences, Najran University, Najran, Saudi Arabia; ^2^Department of Physiotherapy, Asian Institute of Medical Sciences, Sector-21A, Faridabad, Haryana, India; ^3^Department of Physiotherapy, Prakash Institute of Physiotherapy Rehabilitation and Allied Medical Sciences, Chaudhary Charan Singh University (Meerut), Uttar Pradesh, India; ^4^Rehabilitation Research Chair, College of Applied Medical Sciences, King Saud University, Riyadh 11433, Saudi Arabia

## Abstract

**Purpose:**

This study is aimed at determining the impact of work-related low back pain (LBP) on functional performance and physical capabilities.

**Methods:**

This cross-sectional study included women (*n* = 25, mean age, 38.12 ± 4.59) and men (*n* = 25, mean age, 37.20 ± 5.38) with a history of work-related mechanical chronic LBP who visited our university hospital's outpatient department. All participants were assessed for primary outcomes, including the severity of LBP on rest and on activity, functional performance, and physical capabilities using a numeric pain rating scale (NPRS), Roland-Morris disability questionnaire (RDQ), five-time sit-to-stand test (FTSST), and fifty-foot walk test (FFWT), respectively. Independent *t*-tests compared the scores of the outcomes between groups while Pearson's correlation coefficient identified the correlation between the outcomes' measures at a significance level of 0.05.

**Results:**

With a response rate of 63.29%, a total of fifty participant's data were obtained for the analysis. A comparison between women and men groups highlighted a significant difference in the scores of the FTSST and FFWT; however, there were insignificant differences in the scores of the NPRS at rest, NPRS on activity, and RDQ. The bivariate correlation revealed a highly significant, positive, and moderate correlation between the scores of NPRS at rest and FTSST, NPRS on activity and FTSST, NPRS at rest and FFWT, NPRS on activity and FFWT, FTSST and RDQ, and FFWT and RDQ in the women group. Similarly, there was a significant, positive, and low correlation between the scores of FTSST and RDQ and FFWT and RDQ in the men group.

**Conclusion:**

Work-related chronic LBP affected the physical capabilities of women more than those of men. However, it equally affected the functional performance of all participants in the study. Furthermore, work-related chronic LBP affected the physical capabilities (FTSST and FFWT) and functional performance (RDQ) of women more than those of men.

## 1. Introduction

Low back pain (LBP) is defined as pain or discomfort on one or either side of the lower back area descending to the buttocks [1, 2]. LBP affects men and women equally, but men are at a greater risk of work-related back pain. The onset of LBP for the first time in adults most commonly occurs between 30 and 50 years of age, which corresponds to the largest proportion (61.69%, 219/355) of the workforce group [1, 3, 4]. Identifying a specific physical injury is difficult in most cases of LBP (approximately 90%), which is why it is termed nonspecific LBP [5, 6]. Structural misalignment/disruption or tissue damage/degeneration, such as spondylolisthesis, retrospondylolisthesis, spondylarthrosis, disc herniations, and canal stenosis, is often considered and explained in the literature as the causes of LBP [7–9]. However, a biopsychosocial approach has been used to determine the underlying mechanism and chronicity of LBP [8, 9]. This model is widely accepted for utilizing a broader perspective than using only a biomedical perspective because a common and frequent transient feeling of pain does not cause an actual problem to many individuals, but it is rather their own and society's perceptions and reactions to that pain that poses the problem [10, 11]. It exposes the close association between biological, psychological, and social contributors/factors and how social and psychological factors can interact with the brain's activities/processes, which creates a pain-brain cycle that influences the health and illness of individuals [4]. Biological factors at the individual level include age, sex, obesity, general health, heavy birth weight (males), smoking, high levels of pain/disability, healthcare provider attitude, education level, and unemployment [11, 12]. Psychological factors contributing to chronic pain in general, but not limited to LBP, are depression, anxiety, posttraumatic stress disorder, fear avoidance, negative thinking, kinesiophobia, inadequate coping tactics, poor self-efficacy, and preexisting somatic symptoms [6, 13, 14]. Social factors related to the consequences of work-related LBP are work truancy, alienation, laws, reward system, and socioeconomic infrastructures [6, 7, 15, 16]. Psychosocial characteristics in the workplace, including workload, control and support, job happiness, and job appraisal, have been shown to predict the progression of debilitating LBP [17, 18]. Other factors considered to be correlated with work-related LBP are hard physical work, working in shifts, heavy lifting, noncoordinated bending, twisting, pulling, and pushing [7, 16, 19]. Occupational or work-related variables, such as physical and psychological factors, as well as their interactions, are thought to be significant predictors of back pain [16, 19]. Pain and depression have a detrimental effect on one's quality of life. and functionality of patients with LBP; even a previous episode of pain can trigger a new episode of pain [11, 18, 20].

The term “mechanical back pain” refers to discomfort that originates in the spine or its supporting tissues. Symptoms arising from nerve root irritation are known as neuropathic back pain. Mechanical discomfort commonly spreads to the upper thigh and the buttocks, but it is less common than radicular pain that extends below the knee. Mechanical causes of back pain, such as muscular strain, are usually worsened by movement and improved by rest. Although episodes of severe LBP are as likely to start during normal activities as they are after mild trauma, a precipitating event can often help pinpoint the source of the pain [21, 22]. Mechanical factors are significant because various forms of mechanical loads are the leading causes of acute disc prolapse and LBP in general [12, 23]. Researchers discovered that mechanical reasons account for 98% of LBP patients, while malignancy, infection, and visceral disease account for only 2%. There is no identifiable etiology for LBP in 85% of cases, and 90% of patients with uncomplicated mechanical LBP recover in 6 weeks, while the other 5% recover in 12 weeks [24]. The emphasis has shifted from impairment to activity and activity limitation in the International Classification of Functioning, Disability, and Health (ICF) [25].

The term “functioning” encompasses all bodily activities, functions, and involvement [26]. A patient's functional capacity or capability is the ability to perform work-related tasks [27]. Patients with chronic low back pain (CLBP) appear to perform less activity and are more disabled because of chronic pain. Questionnaires, physical performance measurements, work loss assessments, and capacity for work assessments can be used to assess disability. The primary goal of pain rehabilitation is to reduce impairment while restoring functional capacity and involvement [28].

Understanding the relationship between components of the biopsychosocial model and impairment is critical for the best diagnosis and therapy for CLBP patients. Distress, sadness, anxiety, dread, self-efficacy, fear avoidance beliefs, coping methods, and cognitive factors have a greater effect on back pain impairment than biological or biomechanical causes [29–31]. Pain is defined as a sensation of unpleasant feeling that indicates potential or actual damage to the body [32]. The influence of pain on a patient's everyday functioning can be measured as disability or reduction in functional performance. Patients with LBP usually have difficulty picking up items from the floor, rising and descending stairs, and walking [33].

Patients with LBP deteriorate not only their physical health, such as muscle strength, flexibility, and mobility, but also their functional status/performance, which prevents them from returning to work and normal activities [34]. Functional performance refers to what a person can do in his/her native surroundings [35]. The impact of pain on a patient's everyday functioning can be measured as disability or reduction in physical function [36]. Some specialized instruments that assess various aspects of functioning can be used to evaluate the physical competence of patients with LBP, such as the sit-to-stand test, 50-foot (15.24 m) walk test, lumbar flexion, five-minute walking test, and timed up and go test. These tasks were intended to demonstrate the level of physical functioning while performing fundamental daily activities [37].

There is much discussion in the literature from a biomechanical point of view [7–11] whether the change in the size of physiological lordosis is a possible cause of LBP [21–24], in addition to work-related factors, mechanical factors, and low back disability [13–20]. The incidence of LBP is alarming in the working population. Approximately 60% of the seated working population have severe back pain at some point in their lives [38]. The influence of LBP on functional performance and physical capability of men and women has received little attention. There is a scarcity of research on self-reported assessments of functional performance and physical capability in patients with work-related LBP, including a sex-wise comparison of these outcome variables. In addition, it is important to know whether there is an association between LBP intensity, functional performance, and physical capability in men and women in clinical practice. Therefore, research is required to determine the effects of work-related LBP on functional performance and physical capabilities of men and women as well as the association between these outcomes.

Therefore, this study is aimed at determining how work-related LBP affects functional performance and physical capabilities of women and men. This study hypothesized that work-related LBP equally affects the functional performance and physical capabilities of women and men. The results of this study will provide knowledge of the sex-wise impact of work-related LBP on functional performance and physical capabilities as well as the association between these outcomes, which will help clinicians to efficiently identify and treat the symptoms.

## 2. Materials and Methods

### 2.1. Study Design

The study was based on a cross-sectional comparative design, which applied descriptive correlation, and is aimed at evaluating and comparing the effect of the severity of work-related LBP on outcomes related to it and determining the association between functional performance and physical capabilities in women and men with LBP.

### 2.2. Participants

Fifty participants were recruited after screening based on the inclusion and exclusion criteria of this study. The inclusion and exclusion criteria were as follows:

#### 2.2.1. Inclusion Criteria

- Women and men with work-related chronic LBP
- Aged between 30 and 45 years
- Exhibited work-related chronic LBP within 6–12 months of first onset of pain
- Willing to participate in this study

#### 2.2.2. Exclusion Criteria

- Participants exhibiting acute or chronic disc herniation/radiating pain
- History of lumbar spine fracture/systemic diseases involving the spine (e.g., tuberculosis/rheumatoid arthritis)
- Those with serious mental illness, postural anomalies/abnormalities, and neurological and vestibular diseases
- Pregnant females
- Uncooperative patients

### 2.3. Setting

The study sample was obtained from the physiotherapy section of the outpatient department of King Saud University medical facility from September 2019 to March 2020. A convenience sampling method was used, and the participants were differentiated into two groups based on sex.

### 2.4. Ethical Considerations

The ethics subcommittee of King Saud University, Saudi Arabia, approved this study (file ID: RRC-2019-18; dated 28-08-2019). The study preserved human rights through ethical research practices and was conducted in compliance with the Helsinki Declaration. All participants submitted a written informed consent form of their own will before the beginning of the study. All participants were aware of the study and the data inclusion for research purposes and were blinded to the allocation of the study groups.

### 2.5. Procedures

Fifty out of seventy-nine participants with chronic mechanical LBP (mean age, 37 years) were included. The participants were allocated to the female and male groups using a convenience sampling method. All participants were screened based on the inclusion and exclusion criteria and were required to return a completed informed consent form before enrollment in the study. The patients underwent a standardized protocol that included medical history evaluation, physical examination, and assessment of work-related LBP outcomes of the clinical prediction rule, pain evaluation, and disability evaluation. Age, sex, education level, occupation, location, nature of participants' symptoms, and number of days from symptom onset were recorded as part of the demographic data. The lumbar spine range of motion, manual muscle testing, pain severity, and low back impairment were tested during the physical examination. Instruments, such as measuring tape, marker, stopwatch, and chair (43 cm in height), were used in this study. The outcome measures included the numeric pain rating scale (NPRS), Roland-Morris disability questionnaire (RDQ), five-time sit-to-stand test (FTSST), and fifty-foot walk test (FFWT), which were used to evaluate pain, functional performance, and physical capabilities. The two assistant physiotherapists who assessed and collected the data from all participants were blinded to the study.

### 2.6. Outcome Measures

#### 2.6.1. Functional Performance

RDQ is reliable and has been widely used as a disability scale for patients with LBP. It consists of 18 items addressing different aspects of function [39, 40]. The patients were provided a score of one point for each of the 18 items on the questionnaire that were ticked. Thus, an individual patient's score could vary from zero (no disability) to 18 (severe disability), and a higher score was associated with lower functional performance. Participants were instructed to answer questions based on their current state at the time of the examination.

#### 2.6.2. Pain Intensity

The 11-point NPRS is often used to measure pain intensity in patients with LBP [41]. The scale is anchored on the left (score of 0) with the phrase “no pain” and on right (score of 10) with phrase “worst imaginable pain.” This has been shown to yield reliable and valid data [42].

#### 2.6.3. Physical Capabilities

The physical capabilities of the participants were assessed using the FFWT and FTSST [43]. In the FTSST, the participant sat in a chair without leaning on the backrest and was instructed to perform five sit-to-stand movements as quickly as possible without using hand support. The assessor tracked how long it took to finish the test [43]. In the FFWT, the patient walked for 25 feet (7.62 meters) before returning to his original position for a total distance of 50 feet (15.24 m). The assessor also tracked how long it took to complete the examination tests (FTSST and FFWT) which were done twice, and the average of the two results was used in the analysis. A 3-minute rest period was provided between the two rounds of each test. Another rest interval of 5 minutes was provided between FTSST and FFWT. FTSST and FFWT showed adequate levels of test-retest reliability and represented interclass correlation coefficient values of 0.89 and 0.96, respectively [37].

Data were collected for all outcome variables and analyzed to observe the correlation between them.

### 2.7. Statistical Analysis

Statistical Package for the Social Sciences (SPSS v.25, IBM Corp. Armonk, USA) was used for the statistical analysis. The outcome measures, including pain intensity at rest and on activity, functional performance, and physical capability, were tabulated for each participant and were denoted by NPRS at rest and NPRS on activity, RDQ, and FTSST and FFWT, respectively, to explain the results of the study. The value of Pearson's correlation coefficient and level of significance (*p* value) for the women and men groups were denoted by *r*_w_, *r*_m_, and *p*_wm_, respectively, to explain the correlation result. These values were tested for statistically significant differences by using an unpaired *t*-test (independent *t*-test). The prevalence estimates were calculated for the entire study population. The correlations between all variables (LBP, NPRS, RDQ, FTSST, and FFWT) were measured using Pearson's correlation coefficient. The correlation coefficient value was interpreted as 0.00 to 0.30 (0.00 to -0.30), negligible/no relationship; 0.30 to 0.50 (-0.30 to -0.50), low/fair degree of relationship; 0.50 to 0.70 (-0.50 to -0.70), moderate/good relationship; 0.70 to 0.90 (-0.70 to -0.90), high/very good relationship; and 0.90-1.00, very high/excellent relationship [44]. The level of significance (*α*) for all statistical analyses was set at *p* < 0.05.

## 3. Results

Fifty participants with a response rate of 63.29% were included in this study. Twenty participants were excluded because they did not meet the inclusion criteria, three did not participate due to time unavailability, and six did not participate without any reason.

### 3.1. Sex-Wise Comparison of the Groups

A significant difference (95% CI, *p* < 0.05) was observed between the women and men groups on comparison of FTSST (*t* = 2.34, *p* = 0.024) and FFWT (*t* = 2.48, *p* = 0.017) scores. However, an insignificant difference (95% CI, *p* > 0.05) was found on comparison of the scores of NPRS at rest (*t* = 1.060, *p* = 0.294), NPRS on activity (*t* = 0.411; *p* = 0.683), and RDQ (*t* = 0.394, *p* = 0.695). The sex-wise comparison of FTSST, FFWT, NPRS at rest, NPRS on activity, and RDQ scores between women and men is described in Table 1.

### 3.2. Correlation between the Outcome Measures in the Total Sample (*N* = 50)

**Figure 1:**
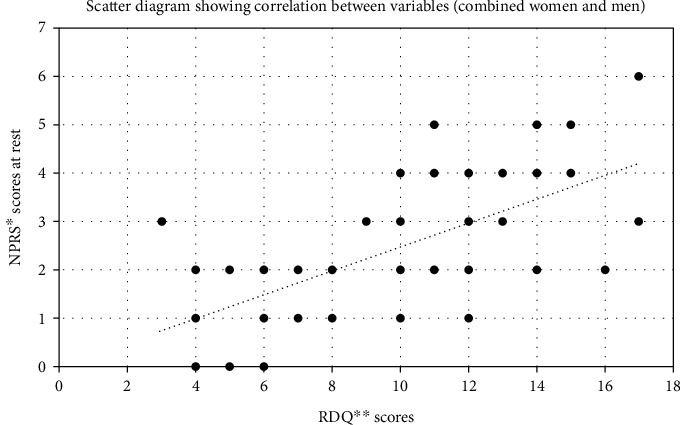
Association (positive linear correlation) of severity of low back pain (NPRS scores) at rest with functional performance (RDQ scores), including women and men participants (*N* = 50). ^∗^NPRS: numeric pain rating scale; ^∗∗^RDQ: Roland-Morris disability questionnaire.

**Figure 2:**
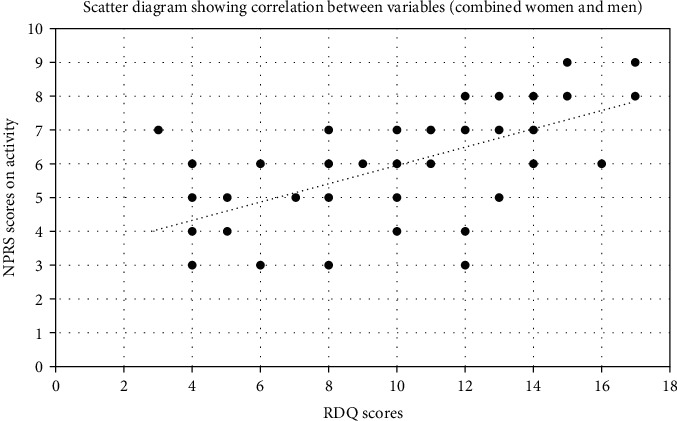
Association (positive linear correlation) of severity of low back pain on activity (NPRS scores) with functional performance (RDQ scores), including women and men participants (*N* = 50). ^∗^NPRS: numeric pain rating scale; ^∗∗^RDQ: Roland-Morris disability questionnaire.

Pearson's correlation coefficient revealed a highly significant, moderate, and positive correlation between the scores of NPRS at rest and RDQ (*p* = 0.001, *r* = 0.665), NPRS on activity and RDQ (*p* = 0.001; *r* = 0.618), NPRS at rest and FFWT (*p* = 0.001, *r* = 0.533), and FFWT and RDQ (*r* = 0.522) (Figures 1 and 2). However, a highly significant, low, and positive correlation was detected between the scores of FTSST and RDQ (*r* = 477, *p* < 0.001), NPRS at rest and FTSST (*p* = 0.001, *r* = 0.437), NPRS on activity and FTSST (*p* = 0.001, *r* = 0.484), and NPRS on activity and FFWT (*p* = 0.001, *r* = 0.393) among all participants (*N* = 50).

### 3.3. Sex-Wise Correlation between the Outcome Measures in Each Group (*n* = 25/Group)

A highly significant, moderate, and positive correlation was found between NPRS at rest and RDQ (*r*_w_ = 0.735, *r*_m_ = 0.619, *p*_wm_ < 0.001) and NPRS on activity and RDQ (*r*_w_ = 0.683, *r*_m_ = 0.571, *p*_wm_ < 0.001) in women and men. Similarly, a highly significant, moderate, and positive correlation was found between NPRS at rest and FTSST (*r* = 0.555, *p* = 0.01), NPRS on activity and FTSST (*r* = 0.651, *p* = 0.001), NPRS at rest and FFWT (*r* = 0.676, *p* = 0.001), NPRS on activity and FFWT (*r* = 0.534, *p* = 0.006), FTSST and RDQ (*r* = 0.561, *p* = 0.004), and FFWT and RDQ (*r* = 0.615, *p* = 0.001) in the women group. In contrast, an insignificant, low, and positive correlation was observed between NPRS at rest and FTSST (*r* = 0.222, *p* = 0.286), NPRS on activity and FTSST (*r* = 0.308, *p* = 0.134), NPRS at rest and FFWT (*r* = 0.275, *p* = 0.183), and NPRS on activity and FFWT (*r* = 0.222, *p* = 0.287) whereas a significant, low, and positive correlation was found between FTSST and RDQ (*r* = 0.434, *p* = 0.030) and FFWT and RDQ (*r* = 0.499, *p* = 0.011) in men. A sex-wise association between the severity of LBP, functional performance, and physical capabilities is shown in Table 2.

## 4. Discussion

The current study performed a sex-wise comparison to reveal the impact of work-related LBP on functional performance and physical capabilities of men and women and observed the relationship between all the outcomes using the NPRS at rest and on activity, RDQ, FTSST, and FFWT.

The results of the current study showed that the correlation between NPRS at rest and RDQ (*r* < 0.665, *p* = 0.0001) and between NPRS on activity and RDQ (*r* < 0.618, *p* = 0.0001) exhibited a moderate degree of correlation among all participants with work-related LBP. The female participants showed a good degree of correlation of NPRS at rest with RDQ (*r* = 0.735, *p* < 0.001) and a moderate degree of correlation of NPRS on activity with RDQ (*r* = 0.683, *p* = 0.002). On the other hand, male participants showed a moderate degree of correlation between NPRS at rest with RDQ (*r* = 0.619, *p* = 0.001) and NPRS on activity (*r* = 0.571, *p* = 0.0029). These results show that pain intensity is closely associated with functional performance in patients with work-related chronic LBP. This means that the higher the pain intensity score, the lower the functional performance level. Many studies have supported our findings, which implies that pain intensity and disability are closely associated in individuals with work-related chronic LBP, and pain-related factors could alter the outcomes of functional performance tasks [37, 45].

The present study found that pain intensity and disability were higher in women than in men. The findings of this study are similar to those of a study conducted on 118 patients with LBP in which women reported higher disability scores because of higher pain intensity than men [46]. This study also showed a significant but weak correlation between the FTSST and NPRS at rest and on activity (*r* = 0.437, *p* = 0.0015; *r* = 0.484, *p* = 0.0004) and between FFWT and NPRS at rest and on activity (*r* = 0.533, *p* = 0.0001; *r* = 0.393, *p* = 0.0048).

The results of the present study showed that physical capability is not significantly affected by pain intensity. This shows a weak correlation; however, it is significant. In a study on 51 patients with LBP, pain intensity was measured with VAS while functional performance was measured with the RDQ. The FFWT, FTSST, and forward bending test were used to measure physical capabilities [30]. It was concluded that there was a weak correlation between pain and physical capabilities and a strong correlation between pain and functional performance [30]. The findings of the study also suggested that low activity levels in individuals with LBP could be due to a fear of not participating in physical activity, such as indoor or job-related activities [30].

The results of this study that compared NPRS at rest and on activity with the FTSST and FFWT showed a moderate degree of correlation with significant results among women. No significant correlations were found among men. This showed that physical capacity is affected by changes in pain intensity, which implies that higher pain intensity caused lower physical capacity, and women were more affected by pain intensity and lower physical capacity than men. The results also showed that pain intensity had no effect on physical capabilities of men. A previous study discovered a modest association between pain and performance tests among patients with chronic LBP and showed that women with chronic LBP had low physical capacity with increased pain intensity [37].

There was a significant moderate correlation between physical capacity and functional performance (RDQ) among participants with work-related LBP (FTSST-RDQ *r* = 0.477, *p* = 0.0005; FFWT-RDQ *r* = 0.522, *p* = 0.0001). Some studies have indicated a moderate association between physical activity and functional impairment in patients with CLBP, implying that those with CLBP and significant degrees of functional disability are also likely to have a low level of physical activity. Another study concluded that self-reported activity limitation and equivalent clinician-measured performance tests had a moderate association. The results showed that physical capabilities decreased in patients with LBP if there were increased levels of disability [47]. A few more studies complemented the results of our study on patients with CLBP using the NPRS, RDQ, and performance tests and found a moderate association between self-reported disability and performance-based assessment of functional disability [37].

In comparison, there was a significantly moderate degree of association between physical capabilities and functional performance of women (FTSST-RDQ *r* = 0.561, *p* = 0.0035; FFWT-RDQ *r* = 0.615, *p* = 0.0011). There was a significant but weak correlation between physical capabilities and functional performance of men (FTSST-RDQ *r* = 0.434, *p* = 0.0303; FFWT-RDQ *r* = 0.499, *p* = 0.011). Based on the findings of the current study, female participants with CLBP had a lower level of physical capability and a greater degree of functional disability. In men, the level of physical capability was not significantly affected by an increase or decrease in the level of disability. One study stated that men have lower pain, better physical capability, and a lower level of disability than women, which could be because they engage in more physical activity at work and have more muscle strength [38]. Men and women may employ different ways of performing daily duties, as men have greater strength than women [48].

Another study reported insignificant results (*p* = 0.5186) when evaluating the influence of LBP with age difference between females (38.12 ± 4.585) and males (37.20 ± 5.385). The results showed that the impact of LBP was the same for males and females irrespective of age [49]. By comparing sex differences and the severity of LBP, the NPRS scores at rest for females and males were 2.60 ± 1.528 and 2.16 ± 1.405, respectively. The NPRS scores on activity for females and males (6.00 ± 1.683 and 5.80 ± 1.756, respectively) showed insignificant results (*p* = 0.2944 at rest, *p* = 0.6828 on activity). The findings of this study showed that there was no association between sex and the impact of LBP. Additionally, it showed that LBP has the same effect on women and men. The results also showed that the pain score was slightly higher in women than in men, but this was not significant. In contrast, one study revealed that there was no association between sex and CLBP, and LBP equally affects men and women [50].

A comparison of functional performance between women (9.88 ± 3.480) and men (9.44 ± 4.369) was performed, and the results were insignificant (*p* = 0.6954). This study showed that the level of disability was the same in women and men. The results of the present study were supported by a previous study involving women and men with LBP and found that there was no significant difference in functional performance [48]. The majority of previous studies that examined the association between physical fitness and functional performance did not differentiate between women and men [51]. The physical capabilities of women and men showed significant results. The results of the FTSST and FFWT showed significant differences in physical capacity. The results of the present study were supported by those of a previous study in which FTSST and FFWT were used and found that men had more physical capacity than women [52]. A group of researchers used clinical performance tests, including the 5 min walk test, FFWT, and FTSST, among women and men in their study and observed that men outperformed women [53]. Men have lower pain scores and better physical capacities than women possibly due to more physical activities at work and greater muscle strength [38].

### 4.1. Limitations

There are certain limitations despite the novelty of this study in terms of sex-wise comparison and examination of some important relationships. This study did not collect employment-related data, such as employment status, compensation status, and involvement of manual tasks; therefore, it did not compare and examine the relationships of these data, which could have made this study more comprehensive and useful to its readers. Furthermore, this study was limited to a small sample size, which cannot be generalized to its relevant global population. However, it paves the way for researchers to advance in the related fields in the future. A bias in the evaluation of disability due to pain could be observed, as any response to the RDQ was based on the individual's own recollection and interpretation while responding to this self-reported questionnaire. The severity of pain measured by the NPRS may also cause a risk of bias in the evaluation of pain at rest and during activity. Some participants in this study took medications that could not be discontinued because of ethical considerations. It is possible that these medications might have affected the severity of their pain. Some participants of the study were working while others were nonworking and could have different mechanical factors causing LBP. Thus, they might have expressed a different opinion regarding their pain, disability, and physical capacity.

Further studies could be beneficial by including a comparatively large population and comparing the physical capability and functional performance between patients with work-related LBP and age-matched normal participants. Additionally, further studies can compare these variables between working and nonworking healthy populations. Similarly, a sex-wise comparison of the different mechanical factors causing LBP between working and nonworking men and women could also be studied.

## 5. Conclusions

The study concluded that the severity of work-related chronic LBP (NPRS at rest/on activity) affects physical capabilities (FTSST and FFWT) of women more than those of men. However, it affects functional performance (RDQ) equally in all participants of the study. The relationship between these outcomes revealed that the severity of work-related chronic LBP was associated with physical capabilities and functional performance of women more than those of men. It could be interpreted that as the severity of work-related LBP increases, physical capabilities and functional performance decrease simultaneously more in women than in men. Therefore, the physiotherapists would have to be able to recognize disparities between perceived functional performance and the actual functional capabilities while treating the patients with work-related chronic LBP. Additionally, the physical therapist must encourage not only gains in physical characteristics, such as strength, flexibility, and mobility, but also improvements in functional status in order for patients to return to employment and normal activities.

### 5.1. Clinical Significance

This study will help to understand how the severity of work-related LBP affects the physical capabilities of patients and their functional performance. It will also help the therapist to make an effective treatment plan that will help improve patients' physical capabilities and will help bring about a remarkable change in ergonomic advises to counter work-related LBP.

## Figures and Tables

**Table 1:** Sex-wise comparison between women and men participants for the outcome variables using unpaired *t*-test (mean ± SD).

Sl.	Variables	Women (*n* = 25) Mean ± SD	Men (*n* = 25) Mean ± SD	Unpaired *t*-test		
				∆MD	*t* value	*p* value
01.	Age (yrs)	38.12 ± 4.59	37.20 ± 5.39	0.92	0.65	0.517
02.	NPRS at rest	2.60 ± 1.53	2.16 ± 1.40	0.44	1.06	0.294
03.	NPRS on activity	6.00 ± 1.68	5.80 ± 1.76	0.20	0.41	0.687
04.	RDQ	9.88 ± 3.48	9.44 ± 4.37	0.44	0.39	0.695
05.	FTSST	14.13 ± 4.53	11.44 ± 3.54	2.69	2.33	0.024^∗^
06.	FFWT	17.00 ± 5.04	14.00 ± 3.32	3.00	2.48	0.017^∗^

^∗^Significant value if *p* < 0.05; SD: standard deviation; ∆MD: mean difference; NPRS: numeric pain rating scale; RDQ: Roland-Morris disability questionnaire; FTSST: five-time sit-to-stand test; FFWT: fifty-foot walking test; *n*: number of participants in each group.

**Table 2:** Association of functional performance with physical capability between women (*n* = 25) and men (*n* = 25) participants using Pearson's correlation coefficient.

Sl.	Variables			RDQ	NPRS-R	NPRS-A
01.	Avg. FTSST	Women	*r* value	0.561	0.555	0.651
		*p* value	*p* value	0.003^∗^	0.004^∗^	0.001^∗^
		Men	*r* value	0.434	0.222	0.308
			*p* value	0.030^∗^	0.286	0.134

02.	Avg. FFWT	Women	*r* value	0.615	0.676	0.534
			*p* value	0.001^∗^	0.001^∗^	0.006^∗^
		Men	*r* value	0.499	0.275	0.222
			*p* value	0.011^∗^	0.183	0.287

03.	RDQ	Women	*r* value	1.000	0.735	0.683
			*p* value		0.001^∗^	0.001^∗^
		Men	*r* value	1.000	0.619	0.571
			*p* value		0.001^∗^	0.003^∗^

^∗^Significant value if *p* < 0.05; NPRS-R: numeric pain rating scale at rest; NPRS-A: numeric pain rating scale on activity; RDQ: Roland-Morris disability questionnaire; Avg. FTSST: average five-time sit-to-stand test; Avg. FFWT: average fifty-foot walking test; *n*: number of participants in each group.

## Data Availability

The data set supporting the conclusions of this article is available through the corresponding author upon reasonable request.
